# Prostate Cancer Aggressiveness Prediction Using CT Images

**DOI:** 10.3390/life11111164

**Published:** 2021-10-31

**Authors:** Bruno Mendes, Inês Domingues, Augusto Silva, João Santos

**Affiliations:** 1 Centro de Investigação do Instituto Português de Oncologia do Porto (CI-IPOP), Grupo de Física Médica, Radiobiologia e Protecção Radiológica, 4200-072 Porto, Portugal; inesdomingues@gmail.com (I.D.); j.a.miranda.santos@gmail.com (J.S.); 2 Faculdade de Engenharia da Universidade do Porto (FEUP), 4200-465 Porto, Portugal; 3 Instituto Superior de Engenharia de Coimbra (ISEC), 3030-199 Coimbra, Portugal; 4 IEETA, Universidade de Aveiro (UA), 3810-193 Aveiro, Portugal; augusto.silva@ua.pt; 5 Instituto de Ciências Biomédicas Abel Salazar (ICBAS), 4050-313 Porto, Portugal

**Keywords:** prostate cancer, radiomic features, classification, risk stratification, computed tomography

## Abstract

Prostate Cancer (PCa) is mostly asymptomatic at an early stage and often painless requiring active surveillance screening. Transrectal Ultrasound Guided Biopsy (TRUS) is the principal method to diagnose PCa following a histological examination by observing cell pattern irregularities and assigning the Gleason Score (GS) according to the recommended guidelines. This procedure presents sampling errors and, being invasive may cause complications to the patients. External Beam Radiotherapy Treatment (EBRT) is presented as curative option for localised and locally advanced disease, as a palliative option for metastatic low-volume disease or after prostatectomy for prostate bed and pelvic nodes salvage. In the EBRT worflow a Computed Tomography (CT) scan is performed as the basis for dose calculations and volume delineations. In this work, we evaluated the use of data-characterization algorithms (radiomics) from CT images for PCa aggressiveness assessment. The fundamental motivation relies on the wide availability of CT images and the need to provide tools to assess EBRT effectiveness. We used Pyradiomics and Local Image Features Extraction (LIFEx) to extract features and search for a radiomic signature within CT images. Finnaly, when applying Principal Component Analysis (PCA) to the features, we were able to show promising results.

## 1. Introduction

The first described Prostate Cancer (PCa) case goes back to 1853, when John Adams, a surgeon at the London Hospital, followed a histological examination for cirrhosis of the prostate gland. He reported the condition as an orphan disease. In 2020, it was the second most frequent malignancy, with 1.414.259 new cases and responsible for 7.3% of all cancer deaths in men [1].

The prostate gland is part of the male reproductive system and about the size of a walnut. It is located in the pelvis surrounding the prostatic urethra and below the bladder [2]. Histologically and clinically, it is a heterogeneous organ divided into four anatomic regions [3]. The central zone encompasses the ejaculatory duct and is relatively immune to cancer. The main body of the prostate gland is the peripheral zone, located posteriorly to the rectum. Most carcinomas arise here [3]. The transitional zone surrounds the urethra and the anterior fibromuscular stroma is non-glandular with no pathological interest [3].

**Figure 1:**
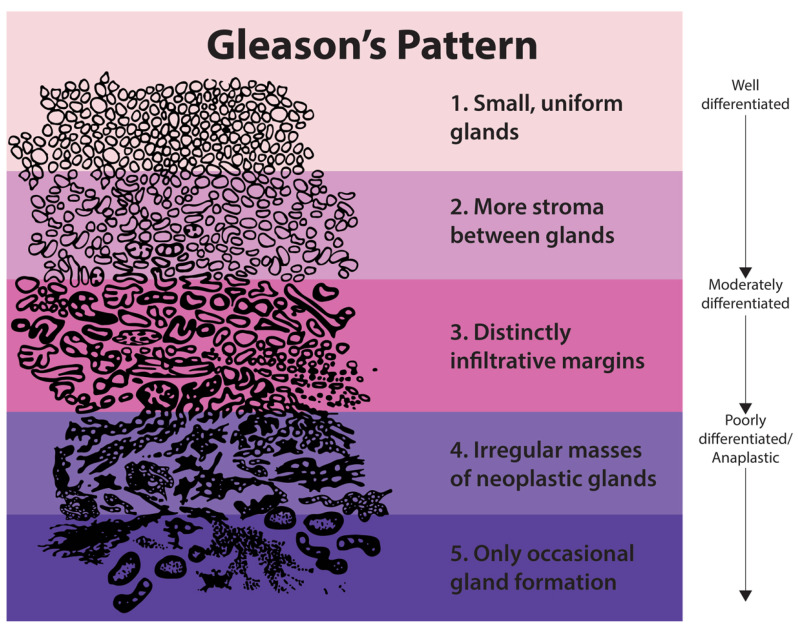
Stratification by Risk Group (RG). Adapted from [5].

PCa is usually asymptomatic at an early stage and screened by Digital Rectal Examination (DRE) and Prostate Specific Antigen (PSA) blood test. The principal method to diagnose PCa is the Transrectal Ultrasound Guided Biopsy (TRUS) with samples taken mainly from the peripheral zone [4]. The pathologist identifies the two most predominant sets of patterns. He then assigns a score of 1 if prostate cells are uniformly packed, up to a grade of 5 depending on pattern irregularity. The sum of both is designated the Gleason Score (GS) and is proportional to PCa aggressiveness. Several studies showed that a GS of 7 = 4 + 3 has the worst prognosis than a GS of 7 = 3 + 4. Taking this into account, Epstein et al. [5] proposed a new stratification by Grade Group (GG), as shown in Figure 1. This new grading system provides the potential to reduce the overtreatment of indolent cancer and reflects the high heterogeneity of PCa [5].

Theoretically GS ranges from 2 to 10 but in practice scores < 6 are never assigned. Table 1 shows the stratification by risk groups.

Grading PCa plays a crucial role in treatment decision outcomes. External Beam Radiotherapy Treatment (EBRT) is a curative option for localised and locally advanced diseases. Also, as a palliative option for metastatic low-volume disease or after prostatectomy for prostate bed and pelvic nodes salvage [6].

In the treatment workflow, patients usually do a Computed Tomography (CT) scan providing the anatomical basics for EBRT planning. In this stage, experts define tumour and tissue-related volumes. According to the International Commission on Radiation Units and Measurements (ICRU) guidelines, Organs At Risk (OARs) are structures or tissues that may suffer morbidity if irradiated. For PCa, the OARs are, by order of priority, rectum, bladder, bowel bag and femoral heads (right and left). The Gross Tumour Volume (GTV) is the gross demonstrable extent and location of the tumour. It may also include regional lymph nodes and distant metastasis if they are indistinguishable from the primary tumour. The Clinical Target Volume (CTV) is a volume that contains a GTV and a margin that reflects the probability of subclinical disease occurrence. The dose prescription is to the CTV plus a clinically acceptable margin that includes organ motion and setup variations (Planning Target Volume (PTV)) [7].

During or after treatment, the tumour marker used to evaluate the effectiveness is the PSA. PSA is an enzyme produced in the prostatic epithelium aiding in the mobility of sperm cells and fertilization. High levels of PSA may indicate the presence of PCa [8], although it may also be associated with BPH, enlarged prostate gland [9]. The traditional PSA level of 4.0 ng/ml is usually the threshold for further evaluation, but this value remains controversial [4,10].

Heterogeneous solid cancers may limit invasive biopsies but open an opportunity to medical imaging. Particularly when significant differences in protein expression patterns proved to correlate to radiographic findings [11]. CT images have a higher spatial resolution than Magnetic Ressonance Imaging (MRI) allowing the evaluation of density, shape and texture characteristics. Radiomics, the extraction of features from radiographic images using data-characterization algorithms, may provide a valuable tool for PCa grading during EBRT. The hypothesis behind radiomics is that quantitative analysis of medical images may provide a similar prognosis power as phenotypes and gene protein signatures.

Most studies seem focused on the initial and diagnosis stage of PCa. Therefore, the prefered imaging modality for radiomic studies is MRI, the *de facto* standard for PCa staging and grading. The present study aims to evaluate the potential use of radiomic features extracted from CT images and provide the baseline for a classifier that predicts PCa aggressiveness during treatment. Such a tool may improve decision outcomes and avoid overdiagnosis and overtreatment.

In this work, we evaluated the extraction of radiomic features from pyradiomics and Local Image Features Extraction (LIFEx) platforms. We searched for a radiomic signature that could predict prostate cancer aggressiveness. However, the lack of characteristic metabolic manifestation of CT proved to be a challenge. Using Principal Component Analysis (PCA) and several variations, we computed Area Under the Receiver Operating Characteristic (AUROC) values using a One-vs-Rest (OvR) Classifier with a linear kernel and obtained promising results.

Following this introductory section, we have Section 2 that presents the state-of-the-art radiomics in prostate cancer. Section 3 hands over the image database and proposed stratification according to the RG. It also shows the proposed method to overcome the lack of CT radiomic signatures for PCa aggressiveness assessment. Section 4 shows the obtained results and grounds the methodology, and finally, Section 5 extends the main conclusions of this work.

## 2. Related Work

PCa diagnosis, staging and grading presents several challenges to overcome. Radiomics, the extraction of quantitative features from medical images using data characterization algorithms, have the ability to provide more relevant information, improving decision outcomes and avoiding overdiagnosis and/or overtreatment.

TRUS is usually used for PCa diagnosis but it may present sampling errors due to the randomness in needle positioning [4]. Besides it is an invasive procedure and can cause complications to the patient such as hematuria, hematospermia and inflammation [12]. The addition of radiomic features to ultrasound images may provide the ability to diagnose PCa without any of these issues. The power of radiomic features to distinguish clinically significant PCa based on ultrasound images has been addressed by Wildeboer et al. [13], Liang et al. [14] with promising results. Liang et al. [14] also added clinical parameters as age, prostate volume, PSA and others. Both studies provided the baseline for deeper analysis using ultrasound images revealing also the potential to use radiomics in an early stage of PCa evaluation.

Multi-parametric Magnetic Ressonance Imaging (mpMRI) is considered the gold standard for PCa assessment. And, with no wonder, most of the radiomic studies found are based on this imaging modality. Prostate Imaging Reporting and Data System (PIRADS) also provides an already established system to enable performance comparisons. In fact, PIRADS 3 score raises some doubts as it defines clinically significant PCa as equivocal. Hou et al. [15] addressed this issue in order to identify clinically significant PCa in PIRADS 3 patients with success. Giambelluca et al. [16] added texture analysis also with PIRADS 3 patients to successfully identify PCa. Chen et al. [17] compared the performance of radiomic-based model with PIRADS. All selected patients had undergone a TRUS and histologically confirmed PCa, GS was available, mpMRI and no prior surgery or EBRT. The same baseline as PIRADS. After statistically selecting six radiomic features the models built from different mpMRI sequences, all outperformed PIRADS predicting PCa.

Biochemical Recurrence (BCR) is also worth mentioning because it is not taken into account by PIRADS. BCR is usually defined as a rise in PSA level after radical prostatectomy or EBRT, although this definition is somewhat controversial. Not all patients with elevated PSA values will develop metastases [18]. In an attempt to predict BCR prior to treatment, Shiradkar et al. [19] identified a set of radiomic features highly predictive of BCR compared to GS, PSA and PIRADS. But the first study to externally validate a radiomics predictive model for high risk PCa with prostatectomy only, was Bourbonne et al. [20] with 88 patients from an external institution. The radiomic model based on Apparent Diffusion Coefficient (ADC) maps achieved an accuracy on the external validation dataset of 76% in predicting BCR. In a pioneer study, Bosetti et al. [21] suggested that energy and kurtosis features from Cone Beam Computed Tomography (CBCT) are predictive of BCR with an AUROC of 0.84.

Recently, Providencia et al. [22] has developed a specially designed algorithm to classify hotspots from bone scintigraphy images. They extracted hand-crafted intensity features and used a pretrained Convolutional Neural Network (CNN) for high-level features following a semisupervised approach, claiming an AUROC of 0.66.

Grading can be challenging for radiomic analysis because the endpoint to address aggressiveness derives from histological findings (GS). But Abraham and Nair [23] proved otherwise. Introducing texture features and a Stacked Sparse AutoEncoder (SSAE) for PCa grade group prediction, Abraham and Nair [23] topped the PROSTATEx-2 challenge with a quadratic-weighted kappa score of 0.2772. Introducing peri-tumoral radiomic features for PCa stratification Algohary et al. [24] also achieved great results with an improvement of 3–6% compared to intra-tumoral features alone. Osman et al. [25] was able to distinguish between GS≤6 and GS≥7 with an AUROC of 0.90 and GS 7(3 + 4) versus GS 7(4 + 3) with an AUROC of 0.98 from CT images.

The mentioned previous studies sustain the idea that the addition of radiomics to already well-established guidelines offer benefits. With the phenotypic and predictive power of radiomic features and the wide availability of CT images, we may provide a tool to assess treatment responses, increasing effectiveness and survival rates.

## 3. Materials and Methods

This work is retrospective research that used treatment plans available at Instituto Português de Oncologia do Porto Francisco Gentil (IPO-PORTO). All patients had undergone a CT scan as part of the EBRT treatment and had the GS available. Section 3.1 presents the image database. In Section 3.3 and Section 3.4 are the methods used to extract and select features, and finally, in Section 3.5 the methods used to build the classifier.

### 3.1. The Image Dataset

The image dataset has CT images from 44 patients following a 3-fold GS risk group stratification, as suggested by Epstein et al. [5] and presented in Table 2. All studies ranged from 2015 to 2019 with curative intent, and patients were between 48 and 58 years old. Two 16 slices CT scanners from General Electric, GE Optima 580 and LightspeedRT16, available at the IPO-PORTO, were used, with 2.5 mm slice thickness, 120 Kvp and automatic tube current modulation.

CT images have a higher spatial resolution than MRI, allowing the evaluation of density, shape and texture characteristics. Although they lack characteristic manifestation [26] and seem to be a poor candidate for radiomic feature extraction, their use for volume delineation in the treatment planning stage makes them widely available.

All images were anonymized and had the approval of use from the ethics committee of IPO-PORTO.

### 3.2. Volumes of Interest

**Figure 2:**
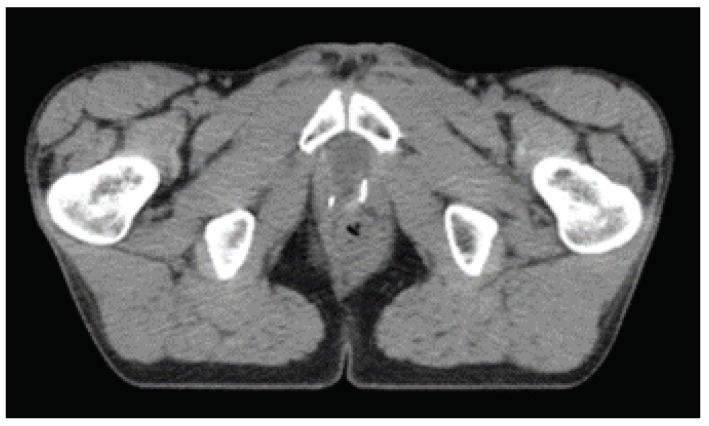
Volumes of interest for prostate adenocarcinoma treatment planning. In orange the ctv; in red the ptv; in green the rectum; the bladder in dark blue and in light blue the femoral heads. Adapted from Gregoire et al. [7].

In EBRT planning, tumor and tissue related volumes are defined by the ICRU. The recommended tool to shape absorbed dose distributions is to define the PTV. Knowledge of uncertainties and variations in tumor volume and machine parameters must be known a priori and thus this volume is very institution dependent. Modern EBRT treatment planning systems use priority rules and weights in the OARs in an optimization framework. The goal is to minimize dose at OARs while preserving the prescribed dose at the PTV [7]. For PCa the OARs are, by order of priority rectum, bladder, bowel bag and femoral heads (right and left). In the treatment planning system dose constrains for each OARs must be taken into account [7]. Figure 2 shows the volumes of interest for prostate adenocarcinoma.

Experts at the institution delineated all Volumes Of Interest (VOIs) and OARs following an ATLAS based semi-automatic approach. The CTV was chosen as the feature extraction region because it contains the most clinical and pathological information.

### 3.3. Feature Extraction

Radiomics are the extracting and quantifying image features in a given volume. Combined with other patient information and clinical outcomes, they can provide a potential tool for decision support models [27]. Radiomics extracts two types of features: semantic and agnostic. Semantic features describe lesions with prognostic values, such as size, shape or necrosis. Agnostic features provide first-order, second-order or higher-order statistics. First-order statistics focus on individual voxels reducing the volume to a single value. Second-order descriptors are texture features grouping voxels with similar statistics and are very useful to measure tumour heterogeneity. Higher-order statistics search for pattern repetitions in the volume [27]. Table 3 shows some of the features that can be extracted.

Features should comply with the Image Biomarker Standardisation Initiative (IBSI), an independent international collaboration that aims at standardizing the extraction of image biomarkers for high-throughput quantitative analysis (radiomics). With this in mind, we used two platforms for feature extraction: PyRadiomics [28], a highly tested and maintained open-source platform, and LIFEx [29], a freeware for radiomic feature calculation in multimodality imaging.

#### 3.3.1. Pyradiomics

Pyradiomics is an open-source python package that allows feature extraction both in 2D or 3D. It is also available as an extension for the 3D Slicer platform [30].

**Figure 3:**
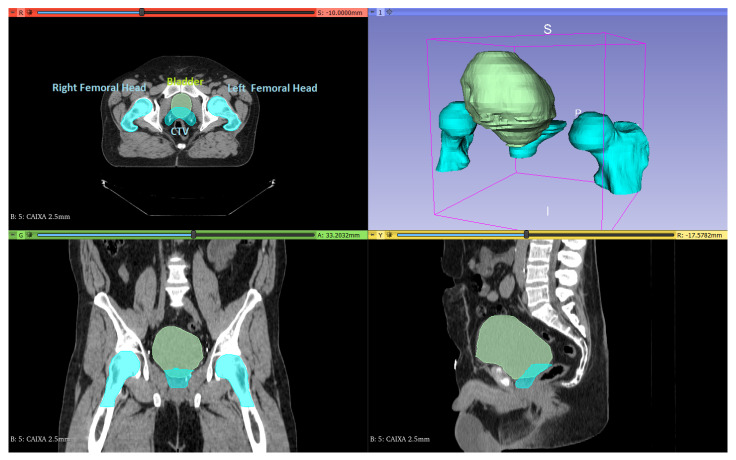
3D Slicer Interface. Visualization of image series, OARs and CTV. **Top-left**: Axial view. **Top-right**: Volumetric reconstruction. **Bottom-left**: Coronal view. **Bottom-right**: Sagittal view.

Figure 3 shows the viewing layout of 3D Slicer. Axial, coronal and sagittal views are perfectly loaded and displayed in the platform as well as a 3D volumetric reconstruction of the OARs and the CTV. All structures and volumes are perfectly registered with the CT series with the z component of every element and planar orientation matching.

Pyradiomics excluded some features due to mathematically similarities to the ones defined in IBSI. For example, the *Sum Variance* and the *Dissimilarity* are identical to the *Cluster Tendency* and *Difference Average* correspondingly [28]. It is important to note that shape descriptors are independent of the grey value and therefore extracted from the label mask. All other features can be retrieved from the original or derived (filtered) masked images. In this work, we did not consider filtered images. Results returned as an ordered dictionary with the unique feature name and additional information on the extraction [28].

#### 3.3.2. LIFEx

LIFEx is a software developed for visualizing multiple imaging modalities and specially designed for feature extractions. It is currently at version 7.1.0 and being actively developed. It presents a very intuitive interface and massive and well-established documentation. It presents a Digital Imaging and Communications in Medicine (DICOM) browser to read images locally or from a network, and even non-DICOM formats are supported. The viewer displays axial, coronal and sagittal slices perfectly aligned and synchronized. A simple drag and drop interface allows to upload structures and desired VOIs [29].

The number of features is smaller when compared to pyradiomics because it is more oriented to Positron Emission Tomography (PET)/CT texture analysis and MRI. In fact, it presents a specific module for PET Standardized Uptake Value (SUV) calculation and another for MRI Perfusion. The results are saved in a csv file for further analysis.

**Figure 4:**
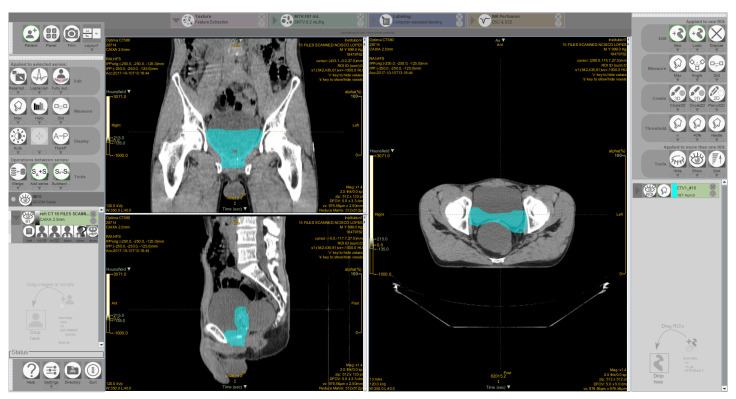
LIFEx Interface. Visualization of image series and ctv in blue.

Figure 4 shows the LIFEx interface displaying axial, coronal and sagittal views. The selected CTV is displayed in blue. The right menu presents several options for segmentation and measuring tools. The left menu with resampling and window-level adjustments and the top menu with all the available feature extraction modules.

For textural analysis, it allows customization of several parameters such as spatial resampling, intensity discretization and rescaling. It is a user-friendly software specially designed for radiomic features studies.

Both platforms, pyradiomics and LIFEx, offer the possibility to extract features from derived images. Wavelets allow overcoming non-rotational invariance. Laplacian of Gaussian (LoG) will emphasize areas of grey level change [28]. In this work, we only used original, non-derived images.

Table 4 summarizes the number of features per feature class possible to extract from pyradiomics and LIFEx.

### 3.4. Dimensionality Reduction

Features were standardized by removing the mean and scaling to unit variance. Each image or volume descriptor represents a point in the feature space. But some are highly correlated, which means overlapped axis. To overcome this issue, we used PCA, which projects the data points to an uncorrelated and orthogonal axis to maximize variance [31]. Dimensionality reduction occurs with the selection of higher variance components.

For this task, we used Scikit-learn, a machine-learning python package [32]. It offers a few variations of PCA, such as linear dimensionality reduction using Singular Value Decomposition (SVD), non-linear dimensionality reduction using kernels (KernelPCA), sparse components that optimally reconstruct data, linear dimensionality reduction using truncated SVD and using the most significant singular vectors to project the data to a lower-dimensional space (IncrementalPCA) [32]. We tried all of these options searching for a combination that would maximize performance.

### 3.5. Model Building and Classification

The adopted methodology allows having a dataset with multiple image features labelled with a particular output, the GS. CT images are not the *de facto standard* for PCa evaluation, so we attempted a more conservative approach. We used an OvR multiclass strategy with an Support Vector Machine (SVM) as a baseline. With this approach, we fitted one classifier per class against all the others. To assess performance, we computed the AUROC curve. For this particular task, we used the python library Scikit-learn [32].

The model was built considering stratified randomized folds. The folds were made by preserving the percentage of samples for each class and the test size was 20% of all slices or volumes.

Our feature extraction pipeline encompasses several steps:

(a) CT images from EBRT;
(b) Manually delineated segmentation by professional experts;
(c) Feature extraction from pyradiomics and LIFEx.

**Figure 5:**
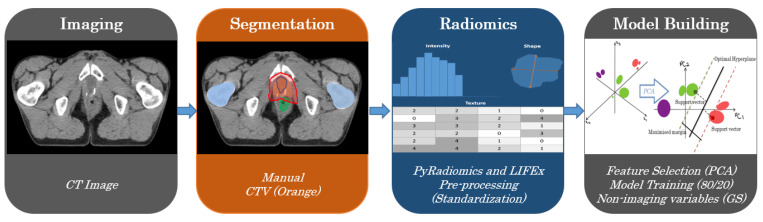
Radiomics Pipeline.

In Radiomic studies, the model is a radiomic signature that relates to a specific clinical endpoint. In our case, such a signature was not possible to find. The model was built with the components obtained from PCA as exemplified in Figure 5.

## 4. Results

CT images are an essential part of EBRT. Although mpMRI shows higher soft-tissue contrast and thus more PCa informative features, this also comes with a higher cost. In this work, we seek to find the potential of CT images for PCa grading and risk stratification.

### 4.1. What Are the More Grade Relevant Features?

The fundamental idea behind radiomics is to find a signature. In other words, a feature or a set of features that shows a high correlation with the GS.

**Figure 6:**
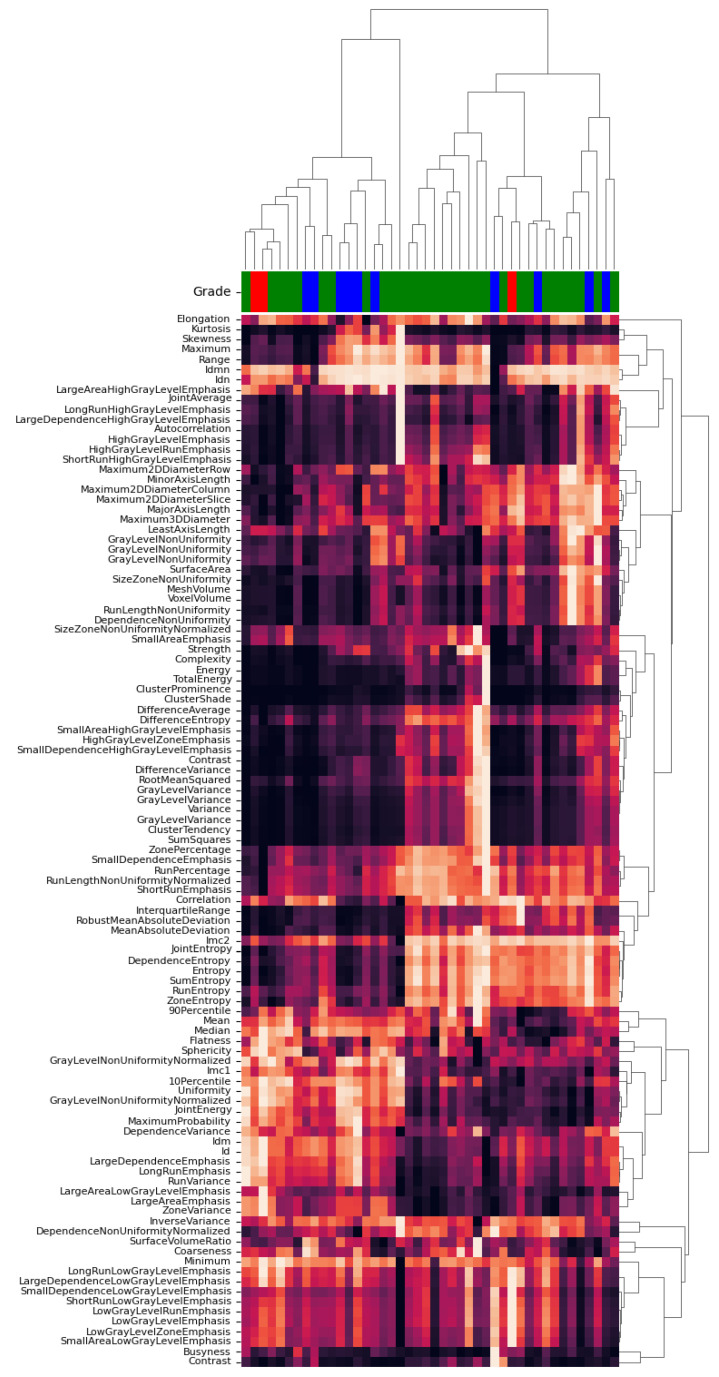
Hierarchical cluster heatmap for pyradiomics features.

**Figure 7:**
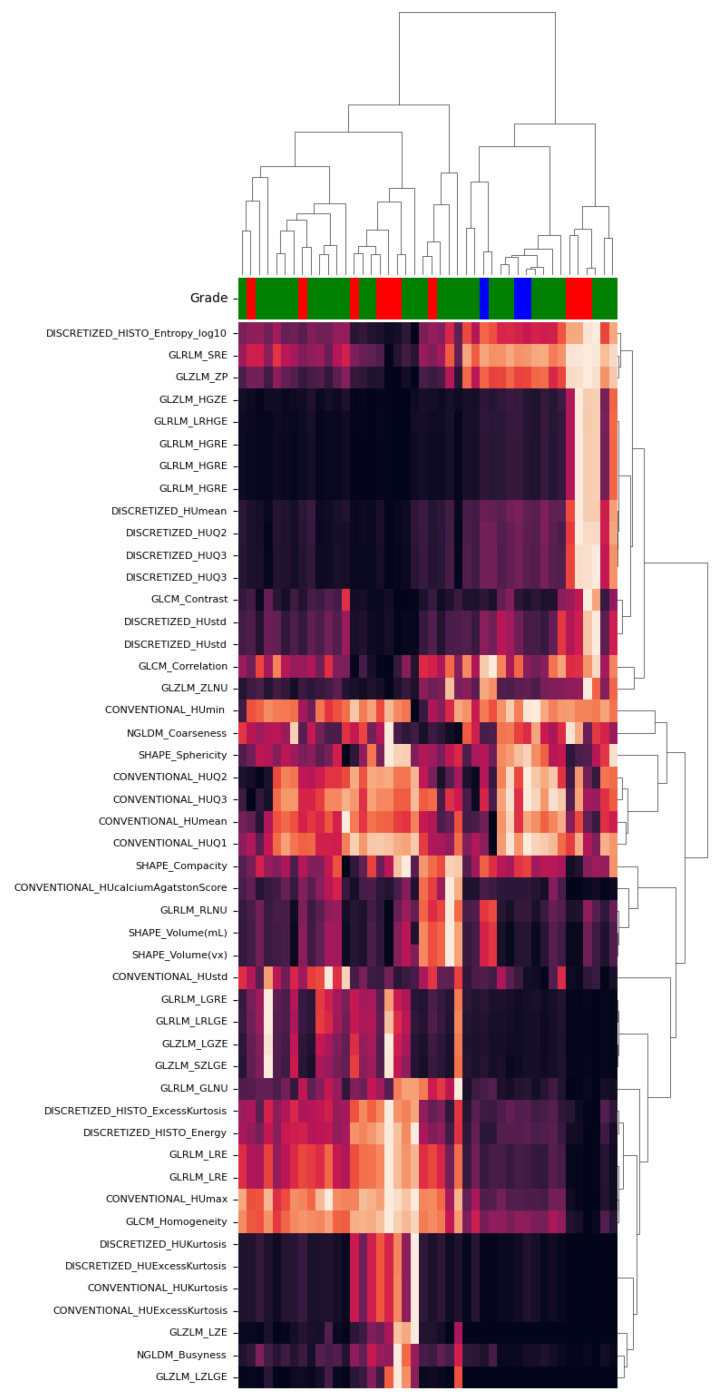
Hierarchical cluster heatmap for LIFEx features.

We computed the heatmaps and dendrograms of all calculated features from pyradiomics and LIFEx. Using the nearest point algorithm and correlation metrics, we clustered all features based on the pairwise distances between observations [32]. Figure 6 and Figure 7 show the obtained hierarchical cluster heatmaps for pyradiomics and LIFEx. The dendrogram reveals a high inter-correlation between features. Also, there is no apparent relation with the RG (represented between the dendrogram and the correlation matrix as Grade). The low soft-tissue contrast and the lack of metabolic manifestation of CT will provide a challenge for a possible radiomic signature.

For classification purposes, a high inter-correlation between features is not a desirable scenario. In radiomic studies, the number of extracted features does not allow adequate interpretability for clinical levels. In this particular case, feature selection seems unfeasible based on the analysis of Figure 6 and Figure 7. There is no apparent pattern considering the clustering by RG following the correlation matrix. Besides, features reveal a low dissimilarity, observed by the close node distance in the dendrogram. To overcome this issue, we tried a dimensionality reduction technique mentioned in radiomic studies [26].

### 4.2. PCA Variations

PCA allows the reduction of dimensionality of large datasets by projecting the most meaningful data to a lower-dimensional space. This reduction may come at the cost of accuracy but, it increases visualization and analysis and faster machine learning algorithms. The sklearn library provides several variations [32]:

- PCA: Linear dimensionality reduction using SVD;
- SparsePCA: Sparse components that can optimally reconstruct original data;
- KernelPCA: Non-linear dimensionality reduction using kernels;
- TruncatedSVD: linear dimensionality reduction by means of truncated SVD;
- IncrementalPCA: Linear dimensionality reduction using SVD but only keeping the most significant singular vectors.

In this work, we explored the differences in performance by computing AUROC values for each of the mentioned PCA methods, as well as the optimal number of components. Sklearn estimates the maximum number of components according to Equation (1).

(1) min((#samples×0.8,#features))

**Figure 8:**
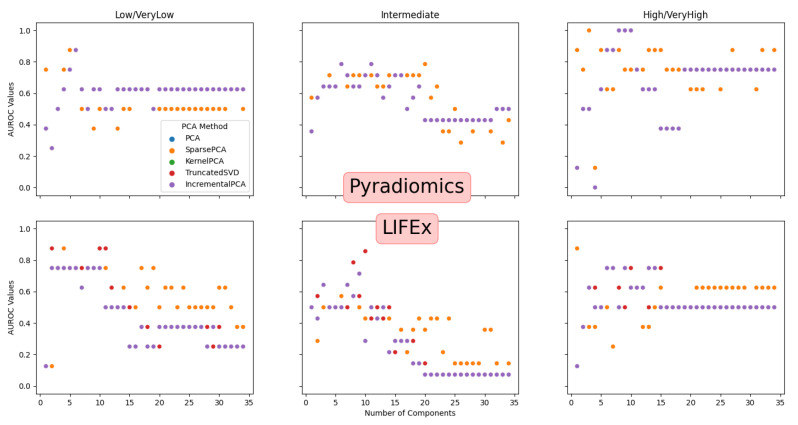
AUROC Values for multiple PCA variations and number of principal components.

For our dataset, the maximum number of components is 34. Figure 8 shows the obtained results for both sets of features (pyradiomics and LIFEx) and for PCA variations and number of components. The obtained values result from 30 runs of the classification pipeline. The multiple OvR classifiers were built with Support Vector Classification and a linear kernel.

Table 5 and Table 6 show the best obtained values. On average, six components are enough to maximize performance (not shown for simplicity). The present study does not attempt to compare pyradiomics and LIFEx but to establish a baseline for deeper studies.

## 5. Discussion

The low soft-tissue contrast and the lack of metabolic manifestation on CT provides a challenge for a radiomic signature. The extracted features, either from pyradiomics or LIFEx, reveal a poor correlation with the RG. The stratification of patients is crucial to decide treatment workflows. The GS is a histological characterization of observed cell patterns. Several studies suggest improvements in the classification framework when adding radiomic intel. With this in mind, we applied several PCA methods, reducing dimensionality by projecting features to a more well-behaved space. The downside is that we lose the ability to identify a radiomic signature for CT to predict PCa aggressiveness.

This study does not intend to be a comparison of pyradiomics and LIFEx. Instead, it intends to be a baseline to provide deeper insights and a classification framework to evaluate EBRT responses.LIFEx seems more PET/CT, and MRI perfusion oriented, offering features that seem more optimized for such imaging modalities. Pyradiomics offers more features and is more imaging modality agnostic. A huge number of features may represent an issue. The results seem better overall using pyradiomics. Also, they seem almost invariant to the PCA method used.

The built models allow the establishment of three classifiers, one for each risk group. In our dataset, the “Low/VeryLow” class is under-represented. This issue will be addressed in the future with the addition of more cases. Also, this is quite an unbalanced dataset considering the distribution of the cases per class.

Both platforms, offer the ability to extract features from derived images, i.e., from wavelets and LoG filters. In our work, we only used the originals. In the future, derived images will be considered and may offer other insights.

## 6. Conclusions

PCa grading is a complex task with multiple variables to be evaluated. The present study provides the baseline to develop an accurate classifier to predict PCa aggressiveness during treatment using CT images. Such a tool may improve decision outcomes and avoid overdiagnosis and overtreatment.

CT images provide a challenge to find a radiomic signature to predict PCa aggressiveness. The application of PCA methods allows the development of a classifier capable of stratifying patients according to the RG.

The well-established guidelines like PIRADS do not take into account a treated prostate. With the biological and morphological changes induced by EBRT, reclassifying or regrading PCa is challenging. With the present study, we do not intend to perform a direct comparison with PIRADS and mpMRI. Instead, we aspire to provide a baseline for a framework capable of reevaluating PCa aggressiveness during treatment. In the EBRT workflow, an initial CT scan is mandatory to provide tissue attenuation coefficients for dose estimations and anatomical intel for volume delineation. The addition of radiomic information can increase the predictive power of CT images. Complemented with the valuable initial findings given by mpMRI, PET/CT and histology, we may walk towards ongoing treatment optimizations. CBCT is also freely available in the EBRT workflow for patient setup verifications. This study may provide the necessary methods to use CBCT as a restaging imaging modality for PCa during treatment.

In the future, we intend to contribute with a clinically implemented system capable of providing valuable intel on the effectiveness of EBRT, improving decision outcomes and survival rates.

## Figures and Tables

**Table 1:** Stratification by risk groups.

Risk Group	Grade Group	GS
Low/Very Low	1	≤6
Intermediate (Favorable/Unfavorable)	2	7 (3+4)
	3	7 (4+3)
High/Very High	4	8
	5	9–10

**Table 2:** Number of cases and images per risk group.

Risk Group	Class	# Cases	# Images
Low/Very Low	0	3	56
Intermediate	1	31	664
High/Very High	2	10	209
**Total**		**44**	** 929**

**Table 3:** Examples of radiomic features.

**First-order**	Mean, Medium, Maximum, Minimum, Entropy, Skewness, Kurtosis, ...
**Second-order**	Autocorrelation, Contrast, Difference Average, Difference Entropy, Inverse Difference Moment, ...
**Higher-order**	Coarseness, Busyness, Complexity, Strength, Gray Level Non-Uniformity, Gray Level Variance, ...

**Table 4:** Pyradiomics and LIFEx feature classes.

Feature Class	# Features	
	Pyradiomics	LIFEx
First Order Statistics	19	12
Shape based	26	4
GLCM	24	6
GLRLM	16	—
GLRM	—	11
NGLDM	—	3
NGTDM	5	—
GLSZM	16	11
GLDM	14	—
**Total**	**120**	** 47**

**Table 5:** Pyradiomics Best AUROC values.

	Low/Very Low	Intermediate	High/Very High
PCA	0.88	0.79	0.88
SparsePCA	0.88	0.79	0.88
KernelPCA	0.88	0.79	0.88
TruncatedSVD	0.88	0.79	0.88
IncrementalPCA	0.88	0.79	0.88

**Table 6:** LIFEx Best AUROC values.

	Low/Very Low	Intermediate	High/Very High
PCA	0.75	0.71	0.75
SparsePCA	0.88	0.64	0.88
KernelPCA	0.75	0.71	0.75
TruncatedSVD	0.88	0.86	0.75
IncrementalPCA	0.75	0.71	0.75

## Data Availability

Not applicable.

## Abbreviations

The following abbreviations are used in this manuscript:

**ADC**	Apparent Diffusion Coefficient
**AUROC**	Area Under the Receiver Operating Characteristic
**BCR**	Biochemical Recurrence
**CBCT**	Cone Beam Computed Tomography
**CNN**	Convolutional Neural Network.
**CT**	Computed Tomography
**CTV**	Clinical Target Volume
**DICOM**	Digital Imaging and Communications in Medicine
**DRE**	Digital Rectal Examination
**EBRT**	External Beam Radiotherapy Treatment
**GG**	Grade Group
**GLCM**	Gray Level Co-occurrence Matrix
**GLDM**	Gray Level Dependence Matrix
**GLRLM**	Gray Level Run Length Matrix
**GLRM**	Grey-Level Run Length Matrix
**GLSZM**	Gray Level Size Zone Matrix
**GS**	Gleason Score
**GTV**	Gross Tumour Volume
**IBSI**	Image Biomarker Standardisation Initiative
**ICRU**	International Commission on Radiation Units and Measurements
**IPO-PORTO**	Instituto Português de Oncologia do Porto Francisco Gentil
**LIFEx**	Local Image Features Extraction
**LoG**	Laplacian of Gaussian
**mpMRI**	Multi-parametric Magnetic Ressonance Imaging
**MRI**	Magnetic Ressonance Imaging
**NGLDM**	Neighborhood Grey-Level Difference Matrix
**NGTDM**	Neighbouring Gray Tone Difference Matrix
**OAR**	Organ At Risk
**OvR**	One-vs-Rest
**PCA**	Principal Component Analysis
**PCa**	Prostate Cancer
**PET**	Positron Emission Tomography
**PIRADS**	Prostate Imaging Reporting and Data System
**PSA**	Prostate Specific Antigen
**PTV**	Planning Target Volume
**RG**	Risk Group
**SSAE**	Stacked Sparse AutoEncoder
**SUV**	Standardized Uptake Value
**SVD**	Singular Value Decomposition
**SVM**	Support Vector Machine
**TRUS**	Transrectal Ultrasound Guided Biopsy
**VOI**	Volume Of Interest
